# Mood Disorders in Young People With Acquired Brain Injury: An Integrated Model

**DOI:** 10.3389/fnhum.2022.835897

**Published:** 2022-06-09

**Authors:** Henrietta Roberts, Tamsin J. Ford, Anke Karl, Shirley Reynolds, Jenny Limond, Anna-Lynne R. Adlam

**Affiliations:** ^1^Psychology, University of Exeter, Exeter, United Kingdom; ^2^Department of Psychiatry, University of Cambridge, Cambridge, United Kingdom; ^3^Department of Psychology, University of Reading, Reading, United Kingdom

**Keywords:** mood disorder, brain injury, young people, mental health, cognition

## Abstract

**Purpose/Objective:**

Young people with paediatric acquired brain injury (pABI) are twice as likely to develop a mood disorder as their peers, frequently have significant unmet socio-emotional needs, and are at over double the risk of going on to use adult mental health services. Recent years have seen significant advances in the development of interventions for young people with mood disorders. However, evidence-based approaches to mood disorders in pABI are lacking and surprisingly little work has evaluated clinical and neuro-developmental models of mood disorders in this population.

**Method:**

We review the literature regarding key mechanisms hypothesised to account for the increased vulnerability to mood disorders in pABI: First, we summarise the direct neurocognitive consequences of pABI, considering the key areas of the brain implicated in vulnerability to mood disorders within a neurodevelopmental framework. Second, we outline five key factors that contribute to the heightened prevalence of mood disorders in young people following ABI. Finally, we synthesise these, integrating neuro-cognitive, developmental and systemic factors to guide clinical formulation.

**Results and Implications:**

We present a framework that synthesises the key mechanisms identified in our review, namely the direct effects of pABI, neurocognitive and neuroendocrine factors implicated in mood and anxiety disorders, maladaptive neuroplasticity and trauma, structural and systemic factors, and psychological adjustment and developmental context. This framework is the first attempt to provide integrated guidance on the multiple factors that contribute to elevated life-long risk of mood disorders following pABI.

## Highlights

- Young people with a brain injury are twice as likely as their peers to develop a mood disorder.
- We review the evidence and identify key mechanisms underpinning this increased vulnerability.
- We present the first integrative framework to guide psychological formulation based on these key factors.
- Clinical formulations should address the effects of brain injury, neurocognitive and neuroendocrine influences on emotional regulation, maladaptive plasticity and trauma, structural and systemic factors, and psychological adjustment and developmental context.

## Paediatric Acquired Brain Injury

Paediatric acquired brain injury (pABI) is a non-degenerative injury to the brain after birth, which can be caused by traumatic (e.g., road traffic accidents) or non-traumatic (e.g., stroke) events. Half of the 1.4 million people per year attending U.K. Accident and Emergency services with a traumatic brain injury are under the age of 15 (National Institute for Health and Care Excellence, 2014). The effects of pABI on social functioning, cognition, emotions, and behaviour make it a leading cause of disability and an important area for investigation (Kassebaum, 2008; Kassebaum et al., 2017). Typically, the longer-term rehabilitative needs of this population are not well supported by service configuration (Royal College of Paediatrics and Child Health, 2003). Young people are often discharged from services following acute treatment, but difficulties arising from pABI may continue to emerge years after injury (e.g., Anderson, 2005) and many young people have significant unmet socioemotional needs (Kurowski et al., 2013; Sariaslan et al., 2016).

Young people with pABI are twice as likely as their peers to develop a common mental health condition such as depression or anxiety (e.g., Kenardy et al., 2012; Max et al., 2012b; Tsai et al., 2014; Laliberté Durish et al., 2018). The worldwide prevalence of depressive disorders in children and adolescents is 2.6%, with 6.5% experiencing an anxiety disorder (Polanczyk et al., 2015). Mood and anxiety disorders are highly comorbid in young people, with estimates suggesting that approximately 30% of anxious adolescents additionally have a depressive disorder, and 72% of adolescents with anxiety and depression had anxiety before they developed depression (Essau, 2003). Following TBI approximately 25% of young people develop a mood disorder, 20% an anxiety disorder, and 13% present with post-traumatic stress disorder (PTSD, Schachar et al., 2015). Pre-existing mental health problems present an additional vulnerability factor for pABI and tend to persist and worsen following injury (e.g., Kurowski et al., 2013; Schachar et al., 2015; Catroppa et al., 2017). Mental health and emotional difficulties have been identified as key clinically modifiable factors associated with quality of life in pABI and are, therefore, important targets for rehabilitation and intervention (Connell et al., 2018). Despite this, there is a lack of evidence-based interventions for mood disorders following pABI (e.g., Gertler et al., 2015) and a framework for formulation and intervention in this population is needed.

Many studies of mental health conditions in the context of ABI, and particularly pABI in young people, acknowledge the challenges associated with the use of standard diagnostic tools in this group (e.g., Kenardy et al., 2012; Laliberté Durish et al., 2018). Young people with pABI may experience injury-related symptoms that are also common to mental health conditions (e.g., loss of energy), and may experience limited insight into and/or difficulties articulating their own emotional experience and associated changes to mood and self-esteem over time. Perhaps as a consequence of this, studies have tended to focus on distinguishing difficulties such as mood and emotional disorders, psychosis, and behavioural difficulties (e.g., Max et al., 1997, 1998) and there is a relative paucity of research untangling the prevalence rates for more fine-grained psychiatric diagnoses within this group.

Another issue to consider in this field, is that mood and anxiety disorders are frequently co-morbid (Garber and Weersing, 2010) and share a number of common underlying processes and mechanisms (Beard et al., 2016). Whilst a number of treatment models have been demonstrated to be highly effective for, for example, mood and anxiety disorders in young people (e.g., cognitive behavioural therapy, Goodyer et al., 2017; Warwick et al., 2017), to our knowledge these have not been systematically tested in young people with pABI. We believe, however, that the existing evidence-base in children and adolescents without an ABI constitutes a promising starting point for adaptation and evaluation of psychological therapies for these conditions in young people with pABI. The focus of this review is, therefore, on addressing these overlapping mood difficulties that are commonly observed in young people with a brain injury, addressing the mechanisms underlying increased vulnerability to these symptoms, and the potential for application and adaptation of common evidence-based treatments for this group.

The effects of pABI are diverse and important for understanding the increased vulnerability to mood disorders in this population. Frontal and limbic regions, and the neuroendocrine stress response are particularly vulnerable in pABI and play a key role in vulnerability to mood disorders (e.g., Cullen et al., 2009; Hagan et al., 2015). In addition, the neurobiological consequences of early life stress and trauma are important to understanding subsequent susceptibility to mental health problems (e.g., Pechtel and Pizzagalli, 2011). Psychological adjustment to the effects of injury, along with structural and systemic factors are also critical to understanding and treating mood disorders in this population.

This review has three aims: First, to identify and summarise the key mechanisms underlying increased vulnerability to mood disorders in young people with pABI. Second, to synthesise these mechanisms and consider their implications for clinical formulation and intervention. Third, to identify future directions and key areas for further research. We first summarise the neurocognitive and emotional consequences of the most common forms of pABI. We then review key factors that may contribute to increased risk for mood disorders following pABI. We summarise outstanding issues and key areas for further investigation. Finally, we synthesise these mechanisms to present a framework for formulation and intervention, and address future directions. The focus of this review is on key clinical and cognitive mechanisms that are implicated in pABI and mood disorders, and there are a number of important socio-economic and demographic factors that were beyond the scope of the present article. We would recommend the following for a discussion of these issues: the prognostic value of demographic characteristics in brain injury (Mushkudiani et al., 2007), epidemiology and outcomes of pTBI (Keenan and Bratton, 2006), social outcomes following pTBI (Yeates et al., 2004), and racial and ethnic differences in outcomes following brain injury (Yeates et al., 2002b; Gary et al., 2009). This article is particularly timely given the context of COVID-19 and the impact that social isolation (e.g., due to lockdown regulations and school closure) can have on feelings of loneliness, and the longer-term consequences on mental health in young people (Barreto et al., 2020; Loades et al., 2020).

## Models to Guide Support for Mood Disorders in Paediatric Acquired Brain Injury

Despite considerable evidence that young people with pABI are at increased vulnerability for mood disorders, there is a lack of models to guide support and interventions for this group. There is now considerable evidence to support adaptations to assessment and therapy for depression and anxiety disorders in adults following brain injury (e.g., Williams et al., 2003a,b; Waldron et al., 2013; Gracey et al., 2016; Ponsford et al., 2016; Simblett et al., 2017; Zelencich et al., 2020). However, there is a relative paucity of such research in young people, which poses challenges for practitioners in adopting an evidence-based framework to mental health assessments and interventions in this population.

We briefly review the evidence suggesting a number of important factors that contribute to heightened vulnerability to mood disorders in young people with pABI: (1) the direct neurocognitive consequences of pABI, (2) the role of neurocognitive and neuroendocrine factors in mood disorders, (3) maladaptive neuroplasticity and the influence of early life stress and trauma on emotion regulation, (4) the influence of structural and systemic factors, and (5) psychological adjustment and the developmental context in which pABI occurs. We seek to integrate these factors into a preliminary framework comprising the first attempt to provide guidance on the multiple factors that contribute to elevated life-long risk of mood disorders in young people with pABI. Our proposed framework provides individual clinicians and multidisciplinary teams with a structured approach to incorporating these different factors within their formulations, and optimising care and outcomes.

## Aetiology and Neurocognitive Consequences of Paediatric Acquired Brain Injury

The most common forms of pABI include traumatic brain injury (TBI, caused by a trauma to the head); encephalopathies, a term that describes any diffuse disease of the brain that causes structural or functional alterations and may result from infection, toxin exposure, metabolic dysfunction, increased intracranial pressure, or lack of blood or oxygen supply to the brain (hypoxic-ischemic and anoxic brain injuries); central nervous system tumours; and stroke. Epidemiological studies indicate that TBI rates range from 70 to 798 per 100,000 persons per year in the age range of 0–14 (Kraus et al., 1991; Hawley et al., 2003; Schneier et al., 2006; Andersson et al., 2010). In the same age group, the reported incidence of stroke varies from 2.1 to 13.0 per 100,000 persons per year, and the incidence of brain tumour varies from 2.8 to 25 per 100,000 persons per year (de Kloet et al., 2013). A multi-centre retrospective hospital study of young people aged 0–24 years in the Netherlands with ABI found that 81.9% were TBIs, with the most common causes being traffic accidents and accidents at home, and 18% had non-trauma causes, with the most common causes being hypoxic-ischemic incidents, and then meningitis/encephalitis, and tumour (de Kloet et al., 2013).

The prefrontal cortex (PFC) and temporal lobes are particularly vulnerable to TBI (Wilde et al., 2005), and to the effects of radiation therapy for brain tumours, which leads to white matter deterioration (Qiu et al., 2007). Damage to the PFC is associated with deficits in goal-directed behaviour, impulsiveness, poor response inhibition, social disinhibition, deficits in emotion recognition, misinterpretation of the moods of others, subjective changes to emotional experience, and difficulties mentalising (Hornak et al., 1996; Bechara et al., 2000; Burgess, 2000; Channon, 2004; Bibby and McDonald, 2005; Henry et al., 2006).

Young people with TBI or pABI following a brain tumour are at greater risk of poorer long-term cognitive, psychological and functional outcomes if they are diagnosed/injured at a younger age (Anderson et al., 2009). Patterns of decline following radiation therapy for brain tumour vary according to age at diagnosis (Palmer et al., 2003), reflecting a reduced rate of acquisition of new skills and knowledge as opposed to a loss of previous learning (Monje et al., 2002; Mabbott et al., 2008). White matter is especially vulnerable to damage from cranial radiation therapy and chemotherapy, particularly during the developmental process of rapid myelination (Moore, 2005). Because younger children begin treatment with a lower white matter volume, this is thought to result in greater deviation from their predicted neurodevelopmental trajectory (Reddick, 2005). For young people diagnosed/injured later in their cognitive development, skills developed before injury (e.g., attentional control) appear to be more resilient than those skills that are still to emerge or consolidate (e.g., cognitive flexibility). Neural growth and myelination that occurs after the injury have been associated with subsequent improvements in executive function following TBI (Casey et al., 2000), but this process is more compromised in more severe injuries which are associated with longer-term difficulties (Beauchamp et al., 2010). Psychological and social difficulties may become more apparent with time as the complexity and demands from the young person’s environment increase (Beauchamp and Anderson, 2013).

Other forms of pABI, such as childhood stroke, also result in a diverse range of neurocognitive and functional impacts determined by individual differences in aetiology, location and lesion volume (O’Keefe et al., 2017). Unlike in adults, where stroke is usually associated with permanent loss of specific functional skills, in young people an altered neurodevelopmental trajectory and subsequent deficits in acquisition of skills is observed (Greenham et al., 2016). As such, the ‘growing into deficit’ model postulates that the extent and severity of subsequent difficulties may emerge over time, reflecting the interplay between impact on subsequent brain maturation and the young person’s environment presenting greater demands (Greenham et al., 2016). Approximately half of young people who have experienced stroke require extra educational support. Again, earlier age of injury and the presence of combined cortical and subcortical lesions are associated with poorer functional outcomes (Mallick et al., 2016; O’Keefe et al., 2017).

Hypoxic and anoxic pABI shows considerable overlap with the effects of other kinds of ABI. However, the selective vulnerability of particular regions of the brain to anoxia also gives some distinctive features to this type of injury. The cerebral cortex (in particular the parietal and occipital lobes), the hippocampus, the basal ganglia and the cerebellum are especially vulnerable (Caine and Watson, 2000; Fitzgerald et al., 2010). When there is also an interruption of blood flow, this can cause damage in the ‘watershed areas’ furthest away from the three major arteries of the brain, which may suffer death of tissue (infarction), like that occurring in a stroke. This can cause specific impairments in episodic memory, social cognition, attention, impulsivity, motor-coordination, visual and auditory perception.

The conditions described above are all examples of the more common forms of pABI, highlighting both a range of differences and commonalities in neurological sequelae across conditions. Functionally, all aetiologies increase the risk of neurological changes affecting multiple brain regions. The neurocognitive consequences of pABI depend on the nature, severity, and location of the injury, age at injury, and factors related to early and subsequent treatment. Certain areas of the brain, in particular the fronto-temporal regions are especially vulnerable and are important to cognitive and emotional functioning. Difficulties may emerge over time as demands on the young person increase and these can be challenging for the young person and family. There are direct neurocognitive effects of pABI on every day functioning which present significant challenges. As we will illustrate below, these brain regions are also implicated in the onset and trajectory of mood disorders. These neurocognitive effects of pABI thus add an additional layer of vulnerability, on top of the socio-emotional challenges of adapting to the physical and psychological effects of experiencing a brain injury.

## Neurocognitive and Neuroendocrine Factors

Neurodevelopmental accounts of mood disorders (e.g., Cullen et al., 2009; Hagan et al., 2015) emphasise the late maturation of aspects of executive functioning in the heightened vulnerability to mood disorders during adolescence. Consistent with this, paediatric depression is associated with deficits in executive control and working memory (Wagner et al., 2014). The direct effects of an injury to fronto-limbic regions (e.g., Max et al., 2012a), and the neurocognitive impact of stress are thus both be important to understanding why young people with pABI are particularly vulnerable to developing a mood disorder.

The PFC does not reach full maturation until early adulthood and it is thought that a neurodevelopmental mismatch between the PFC and relatively matured limbic areas (e.g., the amygdala) that mediate affect may partially explain the increased susceptibility to mental health difficulties during adolescence (Mills et al., 2014; Hagan et al., 2015). Research on ‘sensitive periods’ indicates that exposure to stress may have a greater impact on brain regions that are going through a period of rapid growth (Teicher et al., 2006; Pechtel et al., 2014). It is understood that the prolonged developmental trajectory of complex functions of higher order structures (e.g., frontal lobe structures) makes them particularly vulnerable. Pubertal onset and transition into adolescence is hypothesised to amplify the effect of environmental influences (e.g., stress) on the brain (Tottenham and Sheridan, 2009). Dual process models (e.g., Roiser and Sahakian, 2013; Mills et al., 2014) propose that during adolescence there is a developmental imbalance between ‘hot’ (processing of affect and reward, commonly involving limbic regions such as the amygdala) and ‘cold’ (cognitive processing, including top-down self-regulation arising from frontal structures) brain systems. It is argued that these imbalances interact with genetic and environmental factors to increase vulnerability to affective disorders such as depression (Roiser and Sahakian, 2013), and the impact of a pABI further contributes to this heightened risk for mood disorders.

There is now convergent evidence from over 150 neuroimaging studies that implicate structural and functional abnormalities in regions of the amygdala, hippocampus, subgenual cingulate cortex, and putamen in major depression (see Gray et al., 2020 for a recent meta-analytic review). The medial temporal lobes (including the hippocampus and amygdala) are implicated in models of depression and PTSD, and structural changes to medial temporal brain regions have been demonstrated in depression and PTSD in both adults (e.g., Frodl et al., 2003; Karl et al., 2006) and young people (e.g., MacMillan et al., 2003; Rosso et al., 2005; Karl et al., 2006). Medial network regions, including the amygdala, anterior cingulate, and ventromedial frontal areas, are specifically implicated in the pathophysiology of adolescent depression, with activation of the amygdala most consistently observed across studies of emotional processing, affective cognition, cognitive control, reward processing, and resting state connectivity (Kerestes et al., 2014). The amygdala mediates emotional experiences and emotional memory and is especially important for learning about the emotional significance of stimuli (e.g., safety or danger). Neuroimaging studies indicate a specific role for the amygdala in the processing of fear responses, and emotional engagement with visual stimuli (Phan et al., 2002). It appears to be more reactive in childhood (Tottenham and Sheridan, 2009), as a result, young people have increased susceptibility to emotional experiences (Pechtel et al., 2014), with activity declining into adulthood. Lesions to the amygdala early in life have been linked to exaggerated fear responses and impaired facial expression processing (Tottenham and Sheridan, 2009). The amygdala is regulated by inhibitory feedback from specific areas in the PFC and the pathways connecting the amygdala and PFC develop through adolescence (Tottenham and Sheridan, 2009). The hippocampus is also situated within the temporal lobes and is important to a range of functions including learning and memory consolidation. The anterior portion of the hippocampus provides regulatory feedback to the PFC, amygdala and hypothalamic–pituitary–adrenal axis (HPA axis) and is linked to emotional processing and anxiety. It has been consistently implicated in mood and anxiety disorders, especially depression and PTSD (e.g., Karl et al., 2006; Goodyer, 2008; Tottenham and Sheridan, 2009). The hippocampal effects of childhood stress only emerge in adulthood, which indicates the need for early intervention (Karl et al., 2006; Andersen and Teicher, 2009; Tottenham and Sheridan, 2009).

## Maladaptive Neuroplasticity and Trauma

Early life stress is an established risk factor for subsequent mental health problems (e.g., Green et al., 2014), and the early effects of stress on neural development and brain function are thought to be a key mechanism to understanding this (Tottenham and Sheridan, 2009; Pechtel and Pizzagalli, 2011). In particular, the fronto-limbic circuitry and HPA axis are vulnerable to the effects of injury and early stress (e.g., Tottenham and Sheridan, 2009) and are postulated to be key neurodevelopmental mechanisms in understanding vulnerability to mood disorders in young people (e.g., Cullen et al., 2009; Pechtel and Pizzagalli, 2011).

The HPA axis regulates physical and cognitive responses to stress (Tottenham and Sheridan, 2009; Loman and Gunnar, 2010). It has, therefore, been extensively studied as a biological mechanism implicated in mood disorders (e.g., Yehuda et al., 1991; Goodyer, 2008; Pariante and Lightman, 2008; Sherin and Nemeroff, 2011). HPA functioning may mediate the relationship between early life stress and emotional disorders (e.g., Pariante and Lightman, 2008). Stress and psychological trauma can have a marked impact on the neural structures that support adaptive coping (e.g., ‘neuro-endangerment’; Sapolsky, 2000; Goodyer, 2008; Tottenham and Sheridan, 2009). Early trauma is associated with functional and structural changes in brain circuitries subserving emotion processing and regulation, as well as higher-order cognitive functions such as attention, working memory, cognitive control and creative problem solving (Pechtel and Pizzagalli, 2011). In particular, functional and structural alterations have been reported for the amygdala, the hippocampus, which is susceptible to altered HPA functioning (in particular glucocorticoid feedback loops), and a number of frontal cortical areas involved in emotion regulation, fear extinction (ventromedial PFC; vmPFC), and conflict monitoring (anterior cingulate cortex; ACC) (Driessen et al., 2000; Ovtscharoff and Braun, 2001; Schmahl et al., 2003; Andersen et al., 2008; Goodyer, 2008; Tottenham and Sheridan, 2009). Early life stress is associated with amygdala hyperactivity and growth in children, which may later cause cellular atrophy and have downstream effects on the HPA axis function. This can result in subsequent alterations to the hippocampus which may only be detectable years later (Admon et al., 2009; Tottenham and Sheridan, 2009; Pechtel and Pizzagalli, 2011). Stress additionally alters vmPFC activity, which can lead to impairments in fear extinction learning, enhanced amygdala sensitivity and impaired modulation of amygdala activity (Tottenham and Sheridan, 2009). The PFC is especially vulnerable to the effects of stress, and early stress has been linked to executive functioning deficits, altered development of frontostriatal circuits and poorer inhibitory control (Pechtel and Pizzagalli, 2011). Moreover, executive functioning deficits themselves contribute to increased stress, perpetuating a negative feedback loop.

## Structural and Systemic Factors

Families are likely to experience a process of grief as they adapt to the consequences of pABI and any associated loss of function (e.g., Reed et al., 2015). Families often find it difficult to adjust to the impact of the young person’s injury and taking on a caring role. The clinical literature suggests that adults in a substantial caring role may struggle to adequately meet their own needs and to access appropriate psychological and emotional support (Morris et al., 2016). pABI is established to have a number of significant impacts for families, including high rates of psychological distress, depression and anxiety, enduring burden, social isolation, increased family strain, and loss of income (Rivara et al., 1992; Taylor et al., 2001; Wade et al., 2001, 2004; Yeates et al., 2002a; Anderson et al., 2011; Reed et al., 2015). Families of young people with pABI frequently report significant distress and burden associated with caring for the young person (Aitken et al., 2009), leading to an increased risk of mental health difficulties in parents and siblings, and a breakdown in parental relationships (Taylor et al., 2001; Anderson et al., 2006; Wade et al., 2006; Stancin et al., 2010). Environmental factors are also associated with cognitive and emotional outcomes following pABI, with socio-economic disadvantage and pre-existing family problems associated with poorer outcomes (e.g., Anderson et al., 2009). As a consequence, systemic approaches to neuro-rehabilitation and family-based interventions are recognised as important (Reed et al., 2015). Systemic interventions can incorporate psycho-education, case management, parenting approaches, emotional support, problem solving techniques, and practical adaptations to the young person’s environment (Yeates et al., 2010).

The cognitive and behavioural difficulties associated with pABI are likely to have a substantial impact upon a young person’s functioning in school, and in turn contribute to vulnerability to mood disorders. Young adults with a history of childhood TBI are three times less likely to have finished their school education, and young people with TBI are twice as likely to repeat a school year or have special educational needs (Kinsella et al., 1997; Anderson et al., 2009). The education system involves considerable cognitive and socio-emotional challenges and goals, and schools may be well-placed to contribute to assessment and support for young people with pABI. Schools may be well-placed to detect, and perhaps screen for difficulties and to offer universal and targeted interventions to their pupils (Fazel et al., 2014). Schools might also be well-placed to intervene and mitigate other potential risk factors for depression and anxiety in young people with pABI including, for example, peer relationship difficulties (Yeates et al., 2012). If appropriately resourced schools could contribute to understanding, planning and delivering interventions to support young people with pABI. For example, Ylvisaker and Feeney (2002) work developing context-sensitive interventions to support self-regulation and problem-solving have been demonstrated to be effective in classroom settings (Feeney and Ylvisaker, 2003, 2006; Ylvisaker et al., 2005) and provide a rich framework from which to scaffold additional behavioural interventions.

## Psychological Adjustment and Developmental Context

Relatively little work has focused on the young person’s emotional response and adjustment following pABI, although adaptation and meaning-making have been studied following adult brain injury (Brands et al., 2012; Gracey and Ownsworth, 2012). Goldstein’s (1952) model of adult adaptation to brain injury posits that emotional response to the effects of the injury and loss of functioning through avoidance of perceived catastrophic outcomes are fundamental to approaching rehabilitation and supporting emotional adjustment following ABI. Consistent with this, there is evidence that the experience of a discrepancy between one’s current self and ideal future self predicts emotional outcomes following adult brain injury (Cantor et al., 2005). Cognitive-motivational models of self-regulation (e.g., Carver and Scheier, 1998) predict that negative self-discrepancies initiate repetitive negative thoughts. Worry and rumination are implicated in the onset and maintenance of mental health difficulties such as depression and anxiety (e.g., Nolen-Hoeksema and Watkins, 2011). In young people with pABI, both neuro-cognitive difficulties and the functional impacts of the injury may cause difficulties adapting to the effects of injury, and increase susceptibility to repetitive negative thinking and low mood. Given the key differences between brain injury in adults and in young people (e.g., Beauchamp and Anderson, 2013), it is important to contextualise emotional experience and psychological support within a young person’s family and social context, recognising this as an important foundation for interventions (Limond et al., 2014; Gracey et al., 2015). It is also important to recognise that symptoms of depression and anxiety in pABI may be misattributed to cognitive impairments or personality changes (for example, checking behaviour may be inferred to reflect memory difficulties rather than anxiety).

### Summary and Implications for Paediatric Acquired Brain Injury

There is convergent evidence implicating dysregulation within fronto-limbic circuitry (including the PFC, amygdala, and hippocampus) and the neuroendocrine stress response system (HPA axis) in the pathogenesis of mood and anxiety disorders such as depression and PTSD (Karl et al., 2006; Goodyer, 2008; Cullen et al., 2009; Tottenham and Sheridan, 2009; Pechtel and Pizzagalli, 2011). These biological systems mature during adolescence and is it thought that this may increase both susceptibility to mood disorders and amenability to early intervention during this period in the life cycle (Cullen et al., 2009). Atypical neurodevelopmental trajectories during adolescence, and in particular exaggerated disparities in the relative maturation of frontal and limbic circuitries (with the former being relatively less matured as compared to the latter) may interact with psychosocial and environmental factors to increase vulnerability to mood disorders (Hagan et al., 2015).

In the context of pABI, neurocognitive and other factors can increase anxiety and low mood, as well as untreated symptoms of a mood or anxiety disorder, resulting in additional impairment to cognitive functioning and neurobiological changes that further hinder initial recovery (e.g., Kenardy et al., 2012) and progressively increase susceptibility to future mental health difficulties (e.g., neurodegenerative effects on cortisol regulatory regions as a result of increased HPA activity; Sapolsky, 2001). Early intervention is, therefore, important to reduce the risk of a progressive deteriorating course (Cullen et al., 2009), as well as to help young people to benefit more fully from neuro-rehabilitation. Surprisingly, despite high rates of PTSD (Kenardy et al., 2012) and depression (Laliberté Durish et al., 2018; Ryttersgaard et al., 2020) in TBI, early intervention to address the possible impact of psychological trauma is often overlooked in current practice.

Families report struggling to adapt to the effects of pABI, and systemic and family-based interventions are recognised as playing an important role in rehabilitation. The effects of structural and environmental inequalities may be exacerbated following pABI, and family members are at increased risk of mental health difficulties, and difficulties meeting their own needs, highlighting the need for systemic support.

## Outstanding Issues and Future Directions

An important outstanding question for future research is the relationship between age at injury, neurodevelopmental stage, and age of onset of mood disorders following pABI. In typically developing young people, the greatest increase in incidence of depression occurs during adolescence and early adulthood (Jones, 2013), with 25% of individuals experiencing their first depressive episode before the age of 19 (Kessler et al., 2005; Mokdad et al., 2016). Earlier age of depression onset and a more severe and prolonged first episode is associated with a poorer prognosis (O’Leary et al., 2000; Pettit et al., 2009). As a result, adolescence is identified as a key developmental window in which to intervene in individuals vulnerable to depression (Wagner et al., 2015). Further research is needed to clarify whether a similar pattern is observed in young people with pABI, and the implications of injury at a younger age, and atypical neurodevelopmental trajectories, for the onset and course of mood disorders in differing age groups of young people.

There is evidence from studies with adults and children with mild brain injury that early intervention and psychoeducation results in significant improvements when compared to usual care, including reduced symptoms and lower stress (Ponsford et al., 2001, 2002; Renaud et al., 2020). Children experience critical periods and developmental windows in the acquisition of basic cognitive, emotional, and communication skills. Development also involves many interactions and transactions between domains of function and systems over time, which lead to developmental cascades (changes in the course of development; Cox et al., 2010). Injury-related disruption to the mastery of foundational tasks, therefore, may not only present immediate challenges for that child, but also reduce the possibility to build on those skills in the future as more advanced socio-emotional and cognitive abilities would be expected to develop during adolescence (Limond et al., 2014). A more detailed understanding of the interplay between neurocognitive functioning, age, and clinical presentation, in this population will provide valuable evidence to guide targeted treatment adaptations and inform decision-making regarding the optimal timing of interventions.

A particular consideration when assessing and formulating psychological distress in children and young people with pABI is the implications of emotional disturbances associated with neurocognitive symptoms, such as executive dysfunction, and the complex associations that have been observed between executive functioning, symptoms of mood disorders, and factors such as motivation and participation. Assessments of mood disorder symptoms in pABI may be at increased risk of false positives and false negatives (Williams, 2003), and so including the input of both the child and significant others (e.g., parents, teacher, etc.) is especially important. A particular challenge to assessing symptoms of depression following pABI is distinguishing brain injury related symptoms from similar symptoms arising as a result of depression (e.g., changes to sleep, difficulty concentrating, fatigue, and irritability). Recent developments have been made in the screening and assessment of symptoms of depression in adolescents following brain injury, and there is now good evidence from both adult and adolescent samples to support the use of the PHQ-9 as a screening tool (Fann et al., 2005; Cook et al., 2011; Dyer et al., 2016; Zachar-Tirado et al., 2021).

The measurement and assessment of cognitive and clinical outcomes, and the associations amongst post-injury sequelae across these domains present a number of outstanding challenges for clinicians and researchers in the field. Assessments of the impact and tractor of injury-related changes to cognitive and emotional functioning frequently rely on retrospective accounts of pre-injury status, and the course and presentation of post-injury emotional difficulties prior to the assessment (Max, 2014). There is evidence that structural brain changes and age at injury are associated with novel depressive disorders following pABI (Max et al., 2012a). However, it is not clear whether such structural changes are related to the injury itself, to secondary factors, or pre-existed the injury. A number of studies have found no evidence of an association between injury severity and symptoms of depression, and it has been argued that depression may be a secondary rather than a primary outcome of pABI (Laliberté Durish et al., 2018). However, other studies report an association between injury severity and novel psychiatric disorders (Max et al., 1997), and there is evidence that pre-injury executive functioning, lower income, and poorer family functioning are all associated with greater emotional symptoms and novel mental health diagnoses following pTBI (Max et al., 1997; Ewing-Cobbs et al., 2021). There is substantial evidence that both chronic stress and symptoms of depression are themselves associated with impaired performance on executive functioning tasks, as well as self-reported difficulties with cognitive functioning and memory in everyday life (Burt et al., 1995; Merkelbach et al., 1996; Klimkeit et al., 2011; Baune et al., 2014; Dillon and Pizzagalli, 2018). Greater symptoms of depression or PTSD following pTBI are also associated with poorer cognitive and functional outcomes above and beyond the direct effects of injury severity (O’Connor et al., 2012). As a result, the interplay of emotional sequelae of pABI, performance on neuropsychological tasks, and assessment of functional difficulties in different environments presents a complex and nuanced clinical presentation that requires careful critical appraisal. This will be an important focus for future research and clinical development.

## Treatment Implications

Four clusters of concerns are particularly important in assessment, formulation, and interventions for mood disorders in pABI. First, is the neurological effects of pABI on cognitive and emotional functioning, as outlined above. Second, is the indirect neurodevelopmental effects of early life stress and secondary consequences of brain injury with regard to emotion regulation. The neurodevelopmental context in which these occur, and potential interplay between emergent mood disorder symptoms and executive control is significant in understanding the increased vulnerability to mood disorders following pABI and the importance of early intervention. The third area of concern is the psychological adjustment of the young person and their family following brain injury, and the implications of this for emotion regulation, behaviour and psycho-social functioning. The final area of concern is systemic factors including family/caregiver coping, and the impact of the young person’s injury on their ability to function at school and in peer relationships.

### A Framework for Formulating Mood Disorders in Paediatric Acquired Brain Injury

Limond et al. (2014) proposed psychosocial and systemic aspects of the young person’s context are the foundations required to facilitate successful paediatric neurocognitive rehabilitation. Psychosocial interventions include parental skills training, systemic work, and psychotherapy for mood disorders. This places access to appropriate interventions and support for unmet mental health needs at the heart of supporting families and young people following pABI. We propose a simple framework based on the unique needs and vulnerabilities of this population to guide clinical support and treatment for young people with mood disorder symptoms following pABI.

**FIGURE 1:**
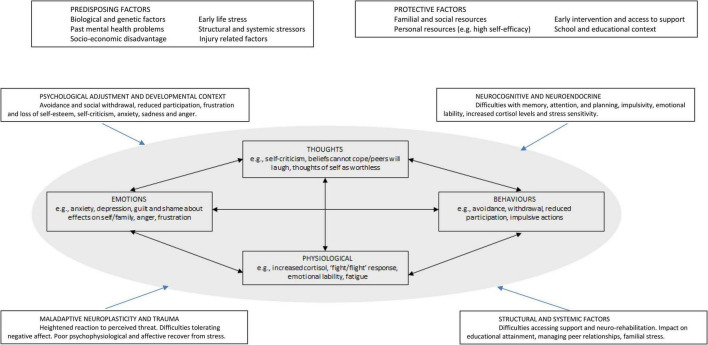
A framework for formulating mood disorders in paediatric acquired brain injury.

Figure 1 draws on cognitive behavioural therapy (CBT) models, including thoughts, feelings, behaviours and physiological responses, to highlight the interaction between pABI specific factors and CBT approaches. It incorporates common examples of the factors outlined above and potential ways to intervene. There is a current lack of evidence-based interventions that specifically address mood and anxiety disorders in pABI (e.g., Gertler et al., 2015). However, this framework identifies key factors to guide the selection and adaptation of existing evidence-based interventions for mood and anxiety disorders in young people (e.g., Weisz et al., 2013). To help adapt standard evidence-based interventions, the figure illustrates how factors specific to pABI sit within a basic cognitive-behavioural formulation of mood disorders. The use of an individual formulation which addresses the individual needs, strengths and challenges experienced by the young person and their family, might help to conceptualise the interplay between neuro-cognitive, psycho-social, and mental health needs. For example, the way in which mental health difficulties manifest themselves as internalising or externalising is likely to be influenced by their neurocognitive profile. Given the range of difficulties and the inter-relationships between them, assessment and intervention for young people with a pABI and mental health difficulties is best provided in a multi-disciplinary team context. It is also important to recognise the significant risk of diagnostic overshadowing and be sensitive to the specific context and challenges in which the young person and their caregiver are operating.

The pre-disposing and protective factors illustrated in Figure 1 highlight the centrality of systemic factors within formulation and intervention for young people with pABI. Interventions for young people with pABI need to be adapted to include greater scaffolding and more explicit support with relevant cognitive skills, as well as psychoeducation about the contribution of pABI to mental health and wellbeing. It may be appropriate to use more frequent, shorter sessions where possible. Many of the emotional, interpersonal and behavioural challenges arising from pABI present themselves in educational, home, and social contexts. The direct neurocognitive and emotional challenges facing young people with pABI, and the contexts in which these challenges are most likely to be experienced, indicate the importance of involving a parent or care-giver in mental health interventions where possible and clinically appropriate.

Selective serotonin reuptake inhibitors (SSRIs) have well-documented efficacy for paediatric depression, which is enhanced when combined with cognitive-behavioural therapy (Giles and Martini, 2016). Antidepressant use is relatively common in adolescents following pABI (Mikkonen et al., 2020). However, there is a paucity of high quality clinical trials of SSRIs following pABI (Max, 2014) and concerns remain regarding insufficient evidence of the long-term effects of pharmacotherapy on the developing brain (Giles and Martini, 2016). Studies of adults experiencing affective symptoms after TBI indicate that serotonergic agents provide the best evidence for treatment of TBI depression, although there is a need for further high-quality randomized controlled trials of psychotropic drug classes (Pangilinan et al., 2010; Yue et al., 2017; Narapareddy et al., 2020) and a recent meta-analysis found no benefit of antidepressant use over placebo for depression following TBI (Kreitzer et al., 2019). Serotonin activity decreases following TBI, and it has been suggested that SSRIs may additionally improve post-injury cognitive functioning by stimulating brain derived neurotrophic factor, resulting in remodelling of the injured brain (Horsefield et al., 2002). There are mixed cognitive effects of SSRIs in clinical trials following pTBI, and studies frequently did not adjust cognitive outcomes for demonstrated concurrent improvements in depression (Writer and Schillerstrom, 2009).

The importance of drawing on a number of theoretical models and approaches to ensure optimal outcomes is recognised for adults following an ABI (Klonoff, 2014) and is likely to be the case for clinical work with young people with pABI. Further research is essential to develop these approaches and guide our understanding of when interventions are best implemented and the specific targets of intervention at different ages and stages of development. This in turn could help guide the development of programmes that can be implemented across healthcare settings, including paediatric and community mental health services where expertise in pABI may be limited. The successful implementation of the proposed framework in to routine clinical practice will require an evaluation of the barriers and facilitators (e.g., Skivington et al., 2021). For example, in the context of COVID-19, there is an increased demand to deliver services via telepsychology. Research suggests that neuropsychological services can be delivered via video-conferencing with web-based resources (adults: Lawson et al., 2020; children and young people: Wade et al., 2020). Other barriers and facilitators include staff training and supervision (in neuropsychology and mental health). Again, virtual (video-conferencing/webinars) training and supervision might enable multiple staff to receive training and supervision at a low cost.

## Conclusion

Young people who survive pABI are at increased risk of developing mood and anxiety disorders, yet evidence-based treatments and service provision are currently lacking. We propose a preliminary framework that seeks to integrate multiple significant factors thought to contribute to the heightened vulnerability in this group: (1) the neurocognitive effects of pABI, (2) the role of neurocognitive and neuroendocrine factors in mood disorders, (3) maladaptive neuroplasticity and trauma, (4) structural and systemic factors, and (5) psychological adjustment and developmental context. Our framework is the first attempt to provide guidance on the multiple factors that contribute to elevated life-long risk of mood disorders in pABI. It provides a structured approach to incorporating these elements within psychological formulations, and optimising care and outcomes. Neurodevelopmental and clinical research highlights the importance of early intervention, and there is considerable potential for the adaptation of cognitive-behavioural approaches. Increased awareness and improved care pathways ways will be a critical first step to supporting this. Schools also have the potential to play an active role in mitigating non-injury related risk factors (e.g., psychological adjustment to the consequences of pABI, peer relationships), identifying mood and anxiety disorders in young people with pABI, and delivering interventions. A proactive multi-professional approach to supporting families and young people is of critical importance to improving the mental health and quality of life of young people following brain injury. In terms of implementation, despite all of the service delivery challenges associated COVID-19, services are also in a unique position to discover their capacity to rapidly respond to change and adapt their practice (e.g., telepsychology). By utilising telepsychology service models, services are also potentially developing low-cost, highly-accessible, and sustainable models of staff training and clinical provision.

## Author Contributions

All authors listed have made a substantial, direct and intellectual contribution to the work, and approved it for publication.

## Conflict of Interest

The authors declare that the research was conducted in the absence of any commercial or financial relationships that could be construed as a potential conflict of interest.

## Publisher’s Note

All claims expressed in this article are solely those of the authors and do not necessarily represent those of their affiliated organizations, or those of the publisher, the editors and the reviewers. Any product that may be evaluated in this article, or claim that may be made by its manufacturer, is not guaranteed or endorsed by the publisher.
